# The impact of electrical stimulation at the back-shu acupoint with an extrinsic diaphragmatic pacing mode on respiratory function and extubation success rate in tracheostomized stroke patients: An observational study

**DOI:** 10.1097/MD.0000000000038595

**Published:** 2024-07-05

**Authors:** Qian Zhang, Yingying Wang, Heng Liu, Huiwei Yang

**Affiliations:** a Department of Anesthesia & Perioperative, Cangzhou Hospital of Integrated TCM-WM Hebei, Cangzhou, Hebei, China; b Baoding Second Central Hospital, Cangzhou, Hebei, China.

**Keywords:** electroacupuncture, external diaphragm pacing, respiratory function, stroke, tracheostomy

## Abstract

To observe of the effect of electrical stimulation at the back-shu acupoint with extrinsic diaphragmatic pacing (EDP) mode on respiratory function and extubation success rate in tracheostomized stroke patients. A total of 200 patients who underwent tracheostomy after a stroke from January 2022 to February 2023 were included in this study. They were divided into 2 groups based on whether electroacupuncture was used: the EDP + electroacupuncture group and the EDP group. We assessed the differences in cough reflex scores and clinical lung infection scores between the 2 groups, and measured levels of blood gas analysis indicators, diaphragmatic function, lung function, maximum inspiratory pressure, and maximum expiratory pressure in both groups. The total effective rate in the EDP + electroacupuncture group was 91.00% (91/100), which was higher than the EDP group’s 80.00% (80/100) (*P* < .05). After treatment, both groups showed a decrease in clinical lung infection scores and cough reflex scores compared to before treatment, with the EDP + electroacupuncture group having lower scores than the EDP group (*P* < .05). After treatment, the pH value, arterial oxygen pressure, and oxygenation index all increased compared to before treatment, with the EDP + electroacupuncture group showing higher values than the EDP group (*P* < .05). After treatment, both groups showed a decrease in arterial carbon dioxide pressure compared to before treatment, with the EDP + electroacupuncture group having lower PaCO_2_ levels than the EDP group (*P* < .05). After treatment, both groups showed an increase in forced vital capacity as a percentage of predicted value (FVC%), diaphragm thickness, diaphragm mobility, maximum inspiratory pressure, maximum expiratory pressure, forced expiratory volume in the first second as a percentage of predicted value (FEV1%), and diaphragm contraction speed compared to before treatment. Additionally, the EDP + electroacupuncture group had higher values in these parameters compared to the EDP group (*P* < .05). The EDP + electroacupuncture group had a shorter average extubation time and a higher extubation success rate compared to the EDP group (*P* < .05). The combination of EDP mode and electroacupuncture at the back-shu acupoint appears to be effective in improving lung function and diaphragmatic function in tracheostomized stroke patients. It also leads to a shorter extubation time and higher extubation success rates.

## 1. Introduction

Stroke is a severe cerebrovascular disorder that poses a significant threat to health, with high mortality and disability rates.^[1]^ Following a stroke, abnormal breathing patterns and respiratory muscle weakness can occur, leading to a reduction in the patient’s ability to cough, clear secretions, and a decline in lung function. This can increase the risk of poststroke pulmonary complications, such as aspiration pneumonia and lung infections, and may even result in patient mortality.^[2]^ To prevent airway obstruction, clinicians often perform a tracheostomy procedure to establish an artificial airway in stroke patients, helping to maintain airway patency and reduce the risk of death.^[3]^ However, some patients may experience difficulties with tube removal, and prolonged tracheostomy tube placement can lead to other complications, significantly impacting the patient’s rehabilitation process.^[4]^ This requires active intervention.

Extrinsic diaphragmatic pacing (EDP) training is a passive respiratory training method that involves the low-frequency electrical stimulation of the phrenic nerve to regulate diaphragm muscle contraction, thereby improving the patient’s respiratory function. It has the advantages of being noninvasive, portable, and easy to operate. Currently, it is widely used in the treatment of patients with tracheostomy after a stroke. However, there are still some patients who experience delayed decannulation even after EDP intervention.^[5]^ Clinical studies have already demonstrated the rehabilitative benefits of acupuncture on limb function in stroke patients.^[6]^ However, there is limited research on the use of acupuncture in improving respiratory function in patients who have undergone tracheostomy after a stroke. This study introduces electroacupuncture into the treatment of tracheostomized stroke patients to observe the impact of electroacupuncture at back-shu acupoints under EDP mode on the respiratory function of these patients and whether it can aid in successful tube removal. The aim is to provide clinical insights and guidance.

## 2. Materials and methods

### 2.1. General data

This study was a retrospective study approved by the Ethics Committee of the Cangzhou Hospital of Integrated TCM-WM. A total of 200 patients who underwent tracheostomy after a stroke from January 2022 to February 2023 were included in this study. They were divided into 2 groups based on whether electroacupuncture was used: the EDP + electroacupuncture group and the EDP group. The EDP group received external diaphragm pacing therapy in addition to standard treatment, while the EDP + electroacupuncture group received electroacupuncture treatment at the back-shu acupoint in addition to standard treatment and EDP therapy. The sample size calculation formula is: *n* = (*Z*^2^ × σ^2^)/*d*^2^, *Z* is the confidence interval, *n* is the sample size, *d* is the sampling error range, and σ is the standard deviation, generally take 0.5. The baseline data of the 2 groups were statistically analyzed (*P* > .05). See Table 1.

**Table 1:** Comparison of 2 groups of baseline data.

Group	n	Gender [n(%)]		Age [(χ̄ ± s), yrs old]	Course of disease [(χ̄ ± s), d]	Stroke type		Comorbidities		
		Male	Female			Ischemic	Hemorrhagic	Hypertension	Diabetes	Hyperlipidemia
EDP group	100	58 (58.00)	42 (42.00)	60.89 ± 8.77	44.58 ± 5.13	56 (56.00)	44 (44.00)	38 (38.00)	26 (26.00)	41 (41.00)
EDP + electroacupuncture group	100	61 (61.00)	39 (39.00)	62.13 ± 9.41	46.01 ± 5.47	51 (51.00)	49 (49.00)	35 (35.00)	29 (29.00)	35 (35.00)
χ^2^		0.187		0.964	1.907	0..503		0.194	0.226	0.764
*P*		.666		.336	.508	.478		.659	.635	.382

### 2.2. Case selection criteria

*Inclusion criteria:* in line with the standards set by the Fourth National Conference on Cerebrovascular Diseases,^[7]^ there is clear imaging evidence to confirm; regardless of gender, age ≥ 60 years old, ≤ 80 years old; pulmonary CT examination showed pulmonary infection; the first onset of the disease, the current vital signs are stable; at the same time, it meets the standard of stroke in the “diagnostic and therapeutic criteria of traditional Chinese medicine”^[8]^: the disease is mainly characterized by hemiplegia, skewed mouth and tongue, dizziness, tongue speechlessness, and partial numbness. Usually, the onset is sudden, combined with the auxiliary diagnosis of tongue, pulse, age, and inducement. Conditional head CT or MRI can be accompanied by abnormal manifestations; Kubota drinking water test ≤ grade 3; the course of disease was 2 weeks to 3 months; indwelling tracheotomy cannula; complete clinical data.

*Exclusion criteria:* with pneumothorax, active pulmonary tuberculosis, wearing a cardiac pacemaker, etc; with severe heart, liver and kidney dysfunction; patients with previous lung disease; the application of ventilator assisted breathing; with malignant tumor or mental illness.

### 2.3. Methods

All patients received comprehensive treatment including nerve nutrition, improvement of cerebral circulation, nutritional support, and rehabilitation training. Additionally, airway interventions such as humidification of the airway and sputum clearance were provided. In the EDP group, patients were positioned in a supine posture, and external diaphragm pacing therapy was administered using the DiaHealth-A variable-frequency portable diaphragm pacing device (Jilin Advantage Kangjian Medical Equipment Co., Ltd). The positive electrode was placed below the sternocleidomastoid muscle, and the negative electrodes were placed on both sides of the pectoralis major muscles. During treatment, blood oxygen saturation and heart rate were monitored. The treatment parameters were as follows: pulse frequency of 40 Hz, pulse amplitude of 70 to 100 V, respiratory rate of 12 to 18 breaths/min, treatment duration of 30 minutes per session, once a day, 5 times a week, for a continuous treatment period of 4 weeks.

In the EDP + electroacupuncture group, in addition to standard treatment and EDP therapy, electroacupuncture was administered at the back-shu acupoint. The EDP treatment method was the same as described previously. For electroacupuncture: Patients were positioned on their sides, and specific acupoints such as bilateral Lung Shu, Spleen Shu, and Kidney Shu were selected. Disposable acupuncture needles (0.25 mm × 40 mm) were used. Standard disinfection of the local skin at the acupoints was performed. The Lung Shu and Spleen Shu points were needled obliquely toward the spine with a depth of 0.5 to 0.8 inches, using a method of even supplement and even draining after obtaining Qi. The Kidney Shu point was needled directly with a depth of 0.8 to 1.2 inches, using a twisting supplement method after obtaining Qi. The needle handle was connected to the SDZ-V electronic acupuncture instrument from Jiangsu Medical Equipment Factory, Huatuo brand, and electroacupuncture was conducted. The dense-sparse wave mode was selected, with a treatment duration of 30 minutes per session, once a day, 5 times a week, for a continuous treatment period of 4 weeks.

### 2.4. Observed indexes

#### 2.4.1. Clinical efficacy criteria

According to the GFC tracheotomy cough reflex grading scale,^[9]^ the total score was 1 to 5 points, of which the score ≥ 2 points indicated the presence of cough reflex disorder, and the score was proportional to the degree of cough reflex disorder. Cured: cough reflex score 1; Effective: cough reflex score decreased by at least 2 points; Effective: cough reflex score decreased by 1 point, but still ≥ 2 points; Invalid: cough reflex score did not change or increase.

#### 2.4.2. *Clinical pulmonary infection score*^[10]^

Including body temperature, white blood cell count, oxygenation, chest X-ray, airway secretions, lung infiltration shadow progress and airway secretions culture. The total score of the scale was 12 points. The higher the score, the more serious the clinical pulmonary infection.

#### 2.4.3. Blood gas analysis

Before treatment and 4 weeks after treatment, 2 mL of radial artery blood was taken from the 2 groups for arterial blood gas analysis. The pH value, arterial oxygen partial pressure (PaO_2_), carbon dioxide partial pressure (PaCO_2_), and oxygenation index (OI) were detected by ABL820 blood gas analyzer of Danish Raydu Company.

#### 2.4.4. Ultrasonic measurement of diaphragm thickness and mobility

Before and after 4 weeks of treatment, ultrasound measurements of diaphragm thickness and mobility were taken with patients in a supine position, breathing spontaneously. A Japanese Canon Apollo i900 ultrasound diagnostic device was used. A linear high-frequency probe was positioned at the junction of the patient’s anterior axillary line or mid-clavicular line with the rib margin to obtain ultrasound images of the diaphragmatic apposition zone. Diaphragm thickness was measured, with 3 respiratory cycles measured and averaged. Diaphragmatic mobility referred to the vertical distance between the lowest and highest points when the diaphragm moved up and down. A low-frequency probe was placed at the junction of the patient’s anterior axillary line or mid-clavicular line with the rib margin. The probe was moved vertically along the cranio-caudal axis of the diaphragm while instructing the patient to breathe calmly. Measurements were taken when diaphragmatic motion was stable and the images were clear. Measurements were also taken after altering the inspiratory capacity. Three respiratory cycles were measured and averaged for each parameter.

#### 2.4.5. Pulmonary function, MIP, maximum expiratory pressure (MEP)

Before and after 4 weeks of treatment, patients’ lung function parameters were assessed using the HI-801 pulmonary function instrument from the Japanese company Jast. Patients were instructed to tightly hold the mouthpiece and perform the deepest inhalation and exhalation with the greatest force and speed possible. The following parameters were recorded: forced vital capacity as a percentage of the predicted value (FVC%), and forced expiratory volume in the first second as a percentage of the predicted value (FEV1%). Additionally, the POWERbreathe K5 testing system was used to measure MIP and MEP. Patients were instructed to perform maximal inhalation after maximal exhalation to measure MIP, and to forcefully exhale after inhalation to measure MEP, with the highest pressure recorded for each.

#### 2.4.6. Extubation situation

The average extubation time and extubation success rate of the 2 groups were recorded.

### 2.5. Statistical processing

SPSS 19.0 was used to process the data. The measurement data of diaphragm thickness and mobility were in normal distribution and the variance was homogeneous. The (χ̄ ± s) method was used to describe the data. The *t* test was used to compare the data before and after treatment. The paired *t* test was used to compare the data before and after treatment. Count data such as stroke type and gender were compared by χ^2^ test, and the number of cases (%) was used to describe. *P* < .05 indicated that there was a statistical difference.

## 3. Results

### 3.1. Comparison of 2 groups of curative effect

The total effective rate in the EDP + electroacupuncture group was 91.00% (91 cases/100 cases), which was significantly higher than the EDP group’s rate of 80.00% (80 cases/100 cases) (*P* < .05). See Table 2

**Table 2:** Comparison of 2 groups of curative effect [n(%)].

Group	n	Cured	Markedly effective	Effective	Ineffective	Total effective rates
EDP group	100	21 (21.00)	37 (37.00)	22 (22.00)	20 (20.00)	80 (80.00)
EDP + electroacupuncture group	100	34 (34.00)	41 (41.00)	16 (16.00)	9 (9.00)	91 (91.00)
χ^2^						4.880
*P*						.027

### 3.2. Comparison of cough reflex score and clinical pulmonary infection score between the 2 groups

The cough reflex score and clinical pulmonary infection score of the 2 groups before treatment were compared (*P* > .05). After treatment, the cough reflex score and clinical pulmonary infection score of the 2 groups were lower than before (*P* < .05), and the cough reflex score and clinical pulmonary infection score in the EDP + electroacupuncture group were lower than those in the EDP group (*P* < .05). See Table 3

**Table 3:** Comparison of cough reflex score and clinical pulmonary infection score between the 2 groups[(χ̄ ± s), scores].

Group	n	Cough reflex score		Clinical pulmonary infection score	
		Before treatment	After treatment	Before treatment	After treatment
EDP group	100	4.02 ± 0.45	1.98 ± 0.26*	8.12 ± 0.91	4.46 ± 0.62*
EDP + electroacupuncture group	100	3.97 ± 0.38	1.33 ± 0.21*	8.03 ± 0.88	2.78 ± 0.45*
*t*		0.849	19.449	0.711	21.929
*P*		.397	.000	.478	.000

Compared with the group before treatment.

* *P* < .05.

### 3.3. Comparison of blood gas analysis indexes between the 2 groups

The blood gas analysis parameters were compared between the 2 groups before treatment (*P* > .05), indicating no significant differences. After treatment, both groups showed an increase in pH value, PaO_2_, and OI compared to before treatment (*P* < .05), and the pH value, PaO_2_ and OI in the EDP + EA group were higher than those in the EDP group (*P* < .05). After treatment, PaCO_2_ in both groups was lower than that before treatment (*P* < .05), and PaCO_2_ in the EDP + EA group was lower than that in the EDP group (*P* < .05). See Table 4

**Table 4:** Comparison of blood gas analysis indexes between the 2 groups (χ̄ ± s).

Group	n	pH value		PaCO_2_ (mm Hg)		PaO_2_ (mm Hg)		OI (mm Hg)	
		Before treatment	After treatment	Before treatment	After treatment	Before treatment	After treatment	Before treatment	After treatment
EDP group	100	7.15 ± 0.12	7.20 ± 0.14*	56.58 ± 5.11	48.12 ± 4.85*	50.58 ± 8.85	65.85 ± 9.03*	181.45 ± 7.89	242.63 ± 12.21*
EDP + electroacupuncture group	100	7.13 ± 0.15	7.29 ± 0.15*	55.87 ± 5.62	44.23 ± 4.51*	51.14 ± 9.06	71.14 ± 8.82*	179.98 ± 8.41	275.69 ± 10.14*
*t*		1.041	4.386	0.935	5.874	0.442	4.191	1.275	20.830
*P*		.299	<.001	.351	<.001	.659	<.001	.204	<.001

Compared with the group before treatment.

PaCO_2_ = carbon dioxide partial pressure, PaO_2_ = arterial oxygen partial pressure, OI = oxygenation index.

* *P* < .05.

### 3.4. Comparison of diaphragmatic function between 2 groups

The diaphragm function parameters were compared between the 2 groups before treatment (*P* > .05). After treatment, both groups showed an increase in diaphragm thickness, diaphragm mobility, and diaphragm contraction speed compared to before treatment (*P* < .05), and the diaphragm thickness, diaphragmatic mobility and diaphragmatic contraction velocity in the EDP + EA group were higher than those in the EDP group (*P* < .05). See Table 5

**Table 5:** Comparison of diaphragmatic function between 2 groups[(χ̄ ± s), cm].

Group	n	Diaphragm thickness		Diaphragmatic mobility		Diaphragm contraction velocity (mm/s)	
		Before treatment	After treatment	Before treatment	After treatment	Before treatment	After treatment
EDP group	100	0.19 ± 0.04	0.21 ± 0.05*	1.93 ± 0.35	2.27 ± 0.38*	10.12 ± 2.25	13.25 ± 2.36*
EDP + electroacupuncture group	100	0.18 ± 0.06	0.24 ± 0.04*	1.90 ± 0.41	2.51 ± 0.41*	9.99 ± 2.17	15.42 ± 2.43*
*t*		1.387	2.967	0.557	4.293	0.416	6.406
*P*		.167	.003	.578	<.001	.678	<.001

Compared with the group before treatment.

* *P* < .05.

### 3.5. Comparison of pulmonary function, MIP and MEP between the 2 groups

The lung function, MIP, and MEP parameters were compared between the 2 groups before treatment (*P* > .05). After treatment, both groups showed an increase in FVC%, FEV1%, MIP, and MEP compared to before treatment (*P* < .05), and FVC%, FEV1%, MIP and MEP in EDP + EA group were higher than those in EDP group (*P* < .05). See Table 6

**Table 6:** Comparison of pulmonary function, MIP and MEP between the 2 groups (χ̄ ± s).

Group	n	FVC%		FEV1%		MIP (cm H_2_O)		MEP (cm H_2_O)	
		Before treatment	After treatment	Before treatment	After treatment	Before treatment	After treatment	Before treatment	After treatment
EDP group	100	62.04 ± 13.24	71.58 ± 12.59*	60.85 ± 10.41	69.86 ± 9.81*	47.12 ± 5.86	56.89 ± 6.33*	63.25 ± 4.25	71.56 ± 6.32*
EDP + electroacupuncture group	100	60.78 ± 14.11	80.79 ± 13.64*	59.82 ± 11.76	77.47 ± 10.15*	46.27 ± 6.08	62.78 ± 5.82*	64.12 ± 4.08	76.89 ± 5.17*
*t*		0.651	4.962	0.656	5.391	1.007	6.850	1.477	6.528
*P*		.516	<.001	.513	.000	.315	.000	.141	<.001

Compared with the group before treatment.

FEV1% = forced expiratory volume in the first second as a percentage of the predicted value, FVC% = the percentage of forced vital capacity to the predicted value, MEP = maximum expiratory pressure, MIP = maximum suction pressure.

* *P* < .05.

### 3.6. Comparison of extubation between the 2 groups

The average extubation time of the EDP + EA group was shorter than that of the EDP group, and the success rate of extubation was higher than that of the EDP group (*P* < .05). See Table 7

**Table 7:** Comparison of extubation between the 2 groups

Group	n	Mean time of extubation [(χ̄ ± s), d]	Success rate of extubation [n(%)]
EDP group	100	27.14 ± 4.53	57 (57.00)
EDP + electroacupuncture group	100	21.32 ± 4.06	10.051
*t*/χ^2^		9.567	4.254
*P*		<.001	.002

## 4. Discussion

Stroke patients often cause airway obstruction due to coma, aspiration, vomiting, tongue drop, and other reasons, respiratory function decreased, respiratory muscle strength decreased to about 50% of normal people.^[11]^ Clinically, tracheotomy is generally used for treatment, and long-term indwelling tracheal cannula is easy to produce a variety of complications, delaying the rehabilitation process.^[12]^ Therefore, early safe extubation is helpful to improve the prognosis. Diaphragm pacing mode in vitro is an important method of pulmonary function rehabilitation in recent years. Diaphragm can produce rhythmic contraction by electrical stimulation of phrenic nerve, thus increasing diaphragmatic activity and muscle fiber endurance.^[13]^ Zhu et al^[14]^ used external diaphragm pacemaker combined with respiratory training to improve the lung function of stroke patients with tracheotomy, which was conducive to early successful extubation.

According to traditional Chinese medicine theory, the essence of a stroke is a combination of deficiency and excess, with an emphasis on excess in the acute phase and deficiency in the recovery phase. The lungs are considered a delicate organ and are susceptible to external pathogens. Respiratory dysfunction after a stroke is often attributed to the stagnation of lung qi. Prolonged illness after a stroke can lead to deficiency of lung and kidney yin, where the insufficient yin fails to transform into qi, resulting in weakened coughing and phlegm-clearing abilities.^[15]^ In the poststroke period, lung infections are closely related to the functions of the lungs, spleen, and kidneys. Back-shu acupoints are located along the Bladder meridian on the back and have the ability to invigorate the body’s yang qi while regulating the internal organs. The “Ling Shu” text in traditional Chinese medicine states, “The back-shu points of the 5 zang organs are located on the back.^[16]^”

In the study by Qiao et al,^[17]^ the use of external diaphragm pacing therapy combined with abdominal muscle electrical stimulation effectively improved the respiratory function of stroke patients. However, their approach did not incorporate the principles of traditional Chinese medicine and meridian theory for selecting acupoints. This current study, based on the pathological characteristics of lung–kidney deficiency in poststroke patients with respiratory dysfunction, selected acupoints such as Lung Shu, Spleen Shu, and Kidney Shu from the back-shu points. The aim was to tonify the earth to overcome the source of phlegm production and nourish kidney water to address the root cause of the stroke, guided by traditional Chinese medicine principles. This study found that the total effective rate in the EDP + electroacupuncture group was higher than that in the EDP group. After treatment, both groups showed a reduction in cough reflex scores and clinical lung infection scores compared to before treatment, with the EDP + electroacupuncture group showing even lower scores than the EDP group. The average extubation time was shorter in the EDP + electroacupuncture group, and the success rate of extubation was higher than in the EDP group. These results suggest that combining external diaphragm pacing with electroacupuncture at back-shu acupoints under this treatment model can enhance the cough reflex in stroke patients with tracheostomy, reduce lung infections, and facilitate successful extubation. This is attributed to the fact that EDP can stimulate the phrenic nerve and improve the work of the inspiratory muscles, thereby enhancing the cough reflex.^[17]^ Internal organ functions are regulated by the autonomic nervous system, and the deep layers of the back-shu acupoints contain sympathetic ganglia. When electroacupuncture stimulates the back-shu acupoints, it produces a microcurrent that stimulates the corresponding segments of the spinal cord’s autonomic nervous system. This helps regulate organ function, enhance the body’s immune system, improve the cough reflex, and facilitate the clearance of pulmonary secretions, thereby preventing or reducing lung infections.^[18]^

Tracheostomy patients often experience poor respiratory function, a decline in lung function, and a state of hypoxia in their bodies. Basic research suggests that lung function is closely related to diaphragm function, as normal diaphragmatic strength helps maintain negative pressure within the chest cavity and lung compliance.^[19]^ However, mechanical ventilation through tracheostomy can lead to progressive atrophy of the diaphragm, resulting in a progressive decrease in diaphragmatic strength. Qiao et al^[20]^ applied acupuncture to back-shu points in combination with diaphragm pacing therapy to treat poststroke respiratory dysfunction. They found that this approach effectively improved the patients’ respiratory function, promoted recovery, and enhanced the Barthel Index. This study observed that after treatment, both groups showed improvements in pH values, PaO_2_, and OI compared to before treatment, with the EDP + electroacupuncture group achieving higher values than the EDP group. Additionally, both groups exhibited a decrease in PaCO_2_ after treatment compared to before treatment, with the EDP + electroacupuncture group having lower values than the EDP group. Furthermore, both groups showed an increase in diaphragm thickness, FVC%, FEV1%, diaphragm mobility, diaphragm contraction speed, MIP, and MEP after treatment compared to before treatment, with the EDP + electroacupuncture group achieving higher values than the EDP group. The results mentioned above suggest that the combination of EDP with electroacupuncture at back-shu acupoints can improve lung function and diaphragm function in stroke patients with tracheostomy, enhance blood gas parameters, and correct hypoxia. This result corroborated the findings of Wei et al in terms of diaphragm movement and blood gas indicators. This is because during EDP intervention, electrical stimulation of the phrenic nerve leads to rhythmic contractions of the diaphragm, resulting in increased muscle fiber size and thickness, and improved diaphragm activity.^[21]^ Electroacupuncture at back-shu acupoints can stimulate the spinal nerves and sympathetic nerves in the corresponding area, generating neurohumoral regulation through a neural reflex mechanism, which in turn regulates organ function and improves both lung and diaphragm function. When these 2 methods are used in combination, they can synergistically enhance their effects through different mechanisms.

This study still has its limitations. The sample size of this study is too small, and the follow-up time cannot observe the long-term curative effect of patients. This study is a retrospective study, and the retrospective study itself may have data bias and confounding, but these do not affect the accuracy of this study. At the same time, the follow-up time was extended to observe the long-term curative effect of the patients. Better explore the effect of electrical stimulation at the back-shu acupoint with an EDP mode on respiratory function.

In summary, the combination of EDP with electroacupuncture at back-shu acupoints can improve lung function and diaphragm function in stroke patients with tracheostomy, enhance blood gas parameters, shorten extubation time, and increase the success rate of extubation.

## Author contributions

**Conceptualization:** Yingying Wang, Qian Zhang, Huiwei Yang.

**Data curation:** Yingying Wang, Qian Zhang, Huiwei Yang.

**Formal analysis:** Yingying Wang, Heng Liu, Qian Zhang, Huiwei Yang.

**Investigation:** Yingying Wang, Heng Liu, Qian Zhang, Huiwei Yang.

**Methodology:** Yingying Wang, Heng Liu, Qian Zhang, Huiwei Yang.

**Writing – original draft:** Yingying Wang, Qian Zhang, Huiwei Yang.

**Writing – review & editing:** Yingying Wang, Qian Zhang, Huiwei Yang.

**Supervision:** Heng Liu.

**Validation:** Heng Liu.

**Funding acquisition:** Huiwei Yang.
